# Non-labeling multiphoton excitation microscopy as a novel diagnostic tool for discriminating normal tissue and colorectal cancer lesions

**DOI:** 10.1038/s41598-017-07244-2

**Published:** 2017-07-31

**Authors:** Takahiro Matsui, Hiroki Mizuno, Takao Sudo, Junichi Kikuta, Naotsugu Haraguchi, Jun-ichiro Ikeda, Tsunekazu Mizushima, Hirofumi Yamamoto, Eiichi Morii, Masaki Mori, Masaru Ishii

**Affiliations:** 1 Department of Immunology and Cell Biology, Osaka University Graduate School of Medicine, 2-2 Yamada-Oka, Suita, Osaka 565-0871 Japan; 2 Department of Gastroenterological Surgery, Osaka University Graduate School of Medicine, 2-2 Yamada-Oka, Suita, Osaka 565-0871 Japan; 3 Department of Pathology, Osaka University Graduate School of Medicine, 2-2 Yamada-Oka, Suita, Osaka 565-0871 Japan; 4 Department of Therapeutics for Inflammatory Bowel Diseases, Osaka University Graduate School of Medicine, 2-2 Yamada-Oka, Suita, Osaka 565-0871 Japan

## Abstract

Multiphoton excitation microscopy (MPM) is regarded as an effective tool that enables the visualization of deep regions within living tissues and organs, with little damage. Here, we report novel non-labeling MPM (NL-MPM) imaging of fresh human colorectal mucosa, which is useful for discriminating cancer lesions from normal tissues quantitatively without any need for resection, fixation, or staining. Using NL-MPM, we visualized three components in human colorectal mucosa, epithelial cells, immune cells, and basement membranes, based on their characteristic patterns of fluorescence. These patterns are characterized by the different auto-fluorescence properties of nicotinamide adenine dinucleotide, nicotinamide adenine dinucleotide phosphate, and flavin adenine dinucleotide and from second harmonic generation (SHG). NL-MPM images were at least as informative to pathologists as were ‘conventional’ images of fixed tissue sections stained with hematoxylin and eosin. Additionally, two quantitative parameters extracted from NL-MPM images – the nucleus diameter (index N) and the intensity of SHG in the basement membrane (index S) – rendered it possible to diagnose cancer regions effectively. In conclusion, NL-MPM is a novel, promising method for real-time clinical diagnosis of colorectal cancers, and is associated with minimal invasiveness.

## Introduction

There is no doubt that malignant tumors remain a serious threat worldwide, often leading to death. With most malignant tumors, accurate diagnosis and early detection are indispensable for improving the treatment outcome and prognosis. Although the ultimate diagnosis of a malignant tumor is made by pathologists, there are some problems with typical pathology-based diagnostic procedures. First, a biopsy obtained for diagnosis is inevitably accompanied by some tissue damage. Second, it takes substantial time to make a diagnosis after biopsy, because various steps are required to prepare pathological specimens from biopsy samples, such as fixation, dehydration, embedding, and staining. For these reasons, the development of a new diagnostic system for malignant tumors for use in real time, involving fewer and less invasive steps, is desirable.

Recent advances in optical imaging technology have enabled observing live phenomena as they occur. Especially, as a major tool for intravital imaging, multiphoton excitation microscopy (MPM) can now be used to observe ‘deep’ regions of the body, such as the brain^1^ and bone marrow cavity^2^ in real time within living animals. The development of new imaging techniques has opened up new paradigms, by applying such techniques to clinical medicine. There are, however, many hurdles to overcome before the clinical application of MPM becomes routine; one such hurdle is the difficulty with labeling cells and tissues *in vivo*, because fluorescent dyes are often toxic, and no genetic labeling is possible in human patients. Thus, it would be desirable to visualize human tissues and organs fluorescently, without the need for additional labeling.

In the present study, we demonstrate a new method of imaging human tissues without labeling to detect cancer cells *in vivo*. We focused on colorectal cancer and analyzed fresh human colorectal mucosa by non-labeling MPM (NL-MPM) imaging. Using MPM imaging for the visualization of fresh, unfixed, and unstained colorectal tissues, we describe our attempt to establish this new diagnostic tool for distinguishing normal and malignant lesions in a quantitative manner, with two indices.

## Results

### NL-MPM technique enables to visualize the histological features of fresh, unstained human colorectal mucosa

**Figure 1:**
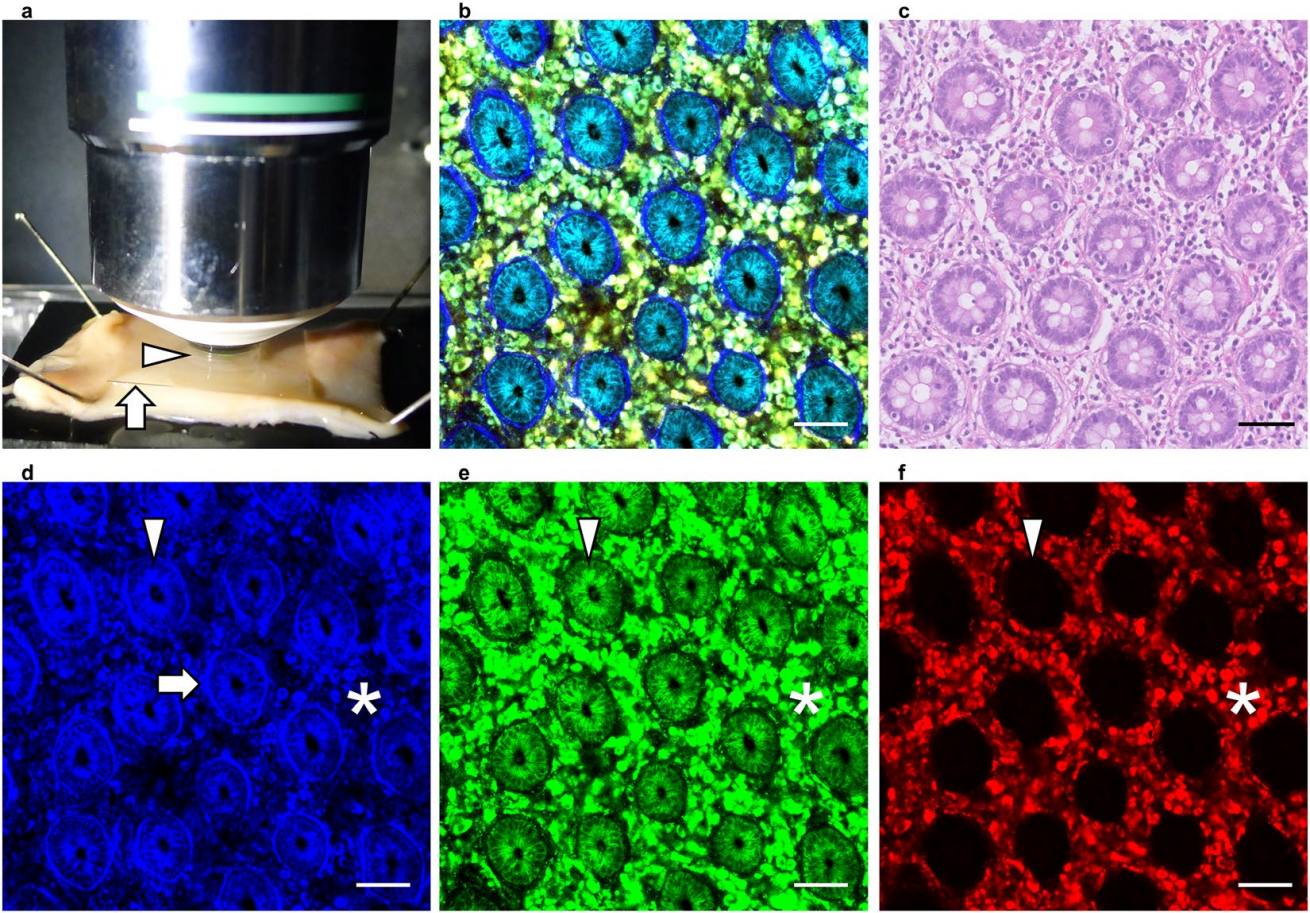
Non-labeling MPM imaging of human normal colorectal mucosa. (**a**) Overview of the imaging approach. Colorectal tissue was placed on a rubber plate with the mucosal surface facing upwards using small pins and was overlaid with a coverslip (arrow) to form a drop of water (arrow head). (**b**) MPM imaging of human normal colorectal mucosa. (**c**) HE staining of normal colorectal mucosa, using the same sample as in (**b**). (**d**–**f**) Images obtained using a 417/60 nm filter (**d**), 480/40 nm filter (**e**), and 629/56 nm filter (**f**) of the samples used in (**b**). Fibrous structures (arrow), epithelial cells (arrow head), and immune cells (asterisk) showed different color patterns. Bar: 50 µm.

We analyzed fresh, unstained normal human colorectal mucosal tissues using an MPM imaging system (Fig. 1a). As shown in Fig. 1b, ductal structures lined up regularly and immune cells in the lamina propria were observed clearly using the procedure. These morphological features, as recognized by MPM imaging, were comparable to those seen using conventional hematoxylin and eosin (HE) staining procedures for fixed sections (Fig. 1c). The MPM imaging data shown consist of three images generated using different band-pass filters. Signals acquired by the 417/60 nm band-pass filter (blue) contain fluorescence derived primarily from ductal epithelial cells and fibrous structures beneath the ducts (Fig. 1d). Fluorescent signals acquired by the 480/40 nm band-pass filter (green) indicate immune cells in the lamina propria and the epithelial cells forming the ductal structures (Fig. 1e). Fluorescence from the 629/56 nm band-pass filter (red) was derived exclusively from immune cells in the lamina propria (Fig. 1f). These data clearly indicate that we can recognize three components independently (epithelial cells, immune cells, and fibrous structures) by their different color patterns in human colorectal epithelial tissue using the NL-MPM procedure.

### Optical background of NL-MPM procedures

**Figure 2:**
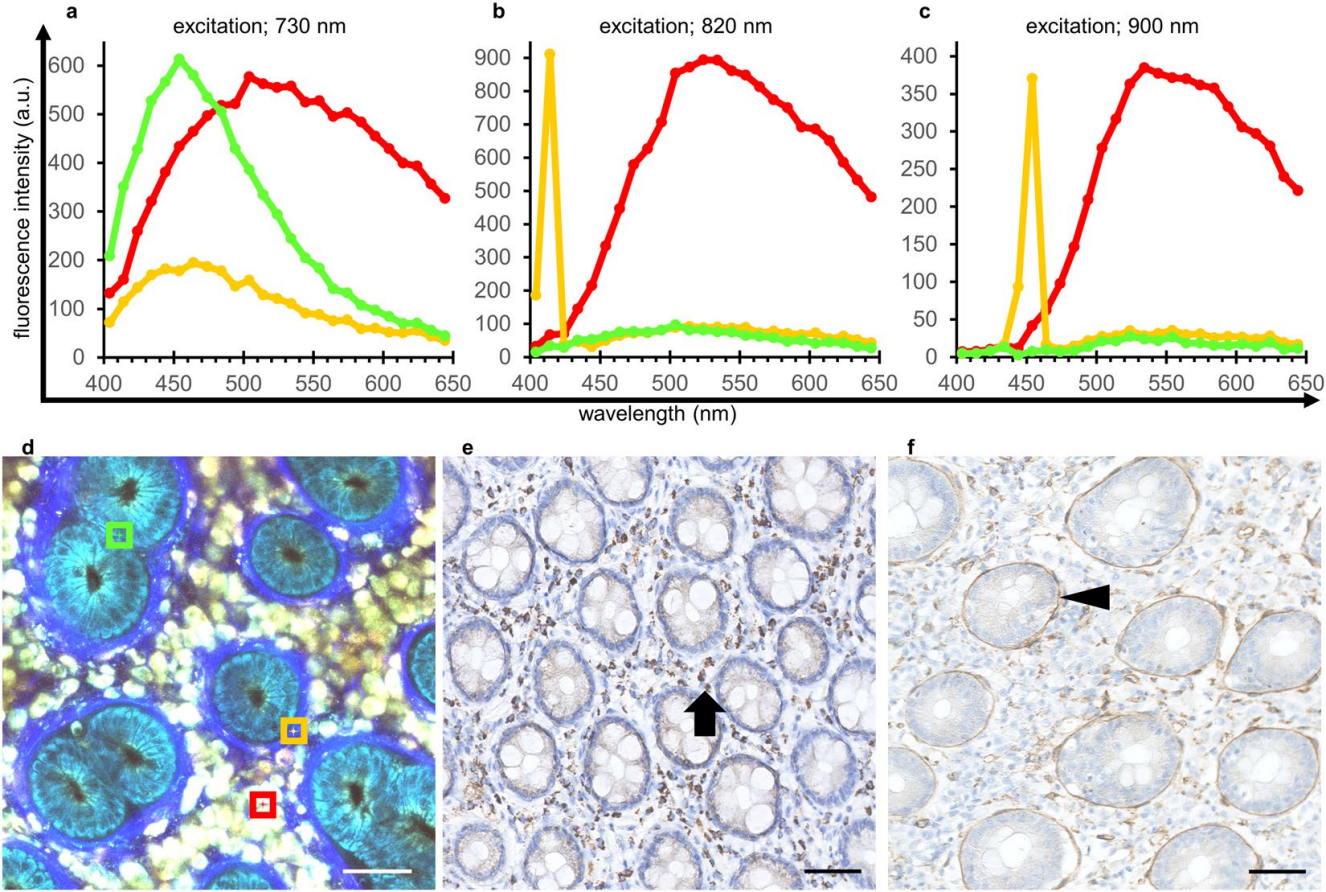
Spectral analysis and immunohistochemical staining of normal colorectal mucosa. (**a**–**d**) Spectral analyses under excitation at 730 nm (**a**), 820 nm (**b**) and 900 nm (**c**) of the ROIs in the MPM image from normal colorectal mucosa (**d**). The green line is the spectrum of the epithelial cell region (green square ROI with a side length of 15 µm in (**d**)); red color indicates the immune cell region (red ROI); and the yellow color indicates the fibrous structure region (yellow ROI). (**e**) Immunohistochemical staining of normal colorectal mucosa using an anti-FAD antibody. Brown staining (FAD positivity) is distributed primarily in immune cells in the lamina propria (arrow). (**f**) Immunohistochemical staining using an anti-laminin antibody. The distribution of laminin-positive staining in the basement membrane (arrow head) is consistent with the fibrous structures observed by MPM imaging derived from SHG signals. Bar: 50 µm.

Next, to characterize the images obtained using the technique, we performed spectral analyses of human colorectal mucosal tissues. We acquired emission signals continuously between 400 and 650 nm with three different excitation wavelengths: 730, 820, and 900 nm (Fig. 2a–d). In epithelial cells, the emission peak was obtained at ~460 nm under 730 nm excitation, but sufficient emission signals could not be obtained under 820 or 900 nm excitation (Fig. 2a–d, green lines). Such characteristics were consistent with those of auto-fluorescence due to reduced pyridine nucleotides: nicotinamide adenine dinucleotide, and nicotinamide adenine dinucleotide phosphate (NAD(P)H)^3^. In immune cells, emission peaks were detected at ~530 nm under all three excitation conditions (Fig. 2a–d, red lines), consistent with the auto-fluorescence signals from flavin adenine dinucleotide (FAD)^3^. In fibrous structures, a sharp and narrow emission fluorescence peak was detected at around half the excitation wavelength at 820 and 900 nm (Fig. 2a–d, yellow lines), indicating typical characteristics of second harmonic generation (SHG), likely due to collagen fibers. Together, these results indicated that different components in human colorectal epithelial tissue showed varying emission spectral patterns that led to color variations within the imaging procedure.

Next, we sought to identify the molecules responsible for these emission signals from the tissues. First, immunohistochemical analyses indicated that FAD was distributed primarily within immune cells in the lamina propria (Fig. 2e), consistent with the emission imaging data from auto-fluorescence possibly caused by FAD (Fig. 2d, red square). We then examined the distribution pattern of laminin, a component of the basement membrane, and compared that with the MPM images. As shown in Fig. 2f, the distribution of fibrous structures, based on the SHG signal, overlapped the laminin-positive areas, demonstrating that the SHG signals originated from beneath the ducts represent the fibrous collagen structures of the basement membranes.

### NL-MPM images are useful for the histopathological diagnosis of colorectal cancer lesions

**Figure 3:**
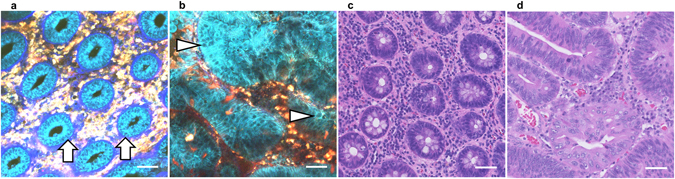
MPM images of human colorectal tissues of different histological statuses. (**a**,**b**) MPM images of normal colorectal mucosa (**a**) and colorectal cancer tissue (**b**). Arrows in (**a**) and arrow heads in (**b**) show the nuclei of epithelial cells. (**c**,**d**) HE-stained images of colorectal mucosa (**c**) and colorectal cancer tissue (**d**), using the same samples as in (**a**) and (**b**), respectively. Bar: 50 µm.

We then performed comparative analyses of normal mucosa and cancer tissues using the procedure. In normal colorectal mucosa, epithelial cells formed regular ductal structures (Fig. 3a). The nuclei of epithelial cells were identified at the side opposite the luminal surface as signal-void regions against a background of fluorescent cytoplasm (Fig. 3a, arrows), consistent with previous reports^4, 5^. In contrast, typical regular ductal formation became obscure in cancer tissue, and the epithelial cells showed irregular-shaped structures with enlarged nuclei (Fig. 3b, arrow heads). These histological features, recognized using the NL-MPM imaging technique, were consistent with conventional HE staining images (Fig. 3c and d), and may offer sufficient information for diagnosis by a pathologist. In fact, NL-MPM images of both normal tissues (*n* = 16) and colorectal cancer lesions (*n* = 20) were examined randomly by two experienced pathologists, who could make correct diagnoses based only on the NL-MPM images (Table 1). These findings indicated that NL-MPM images from the procedure were as useful as conventional HE staining of resected/fixed specimens for differential diagnosis of cancerous versus non-cancerous regions, demonstrating the potential utility of NL-MPM in the clinic.

**Table 1:** Comparison of diagnostic results by pathologists between HE staining and NL-MPM.

		HE diagnosis		
		normal	cancer	total
NL-MPM diagnosis	normal	16	0	16
	cancer	0	20	20
	total	16	20	36

### Nuclear diameter is one of the useful parameters to distinguish cancer from normal tissue

**Figure 4:**
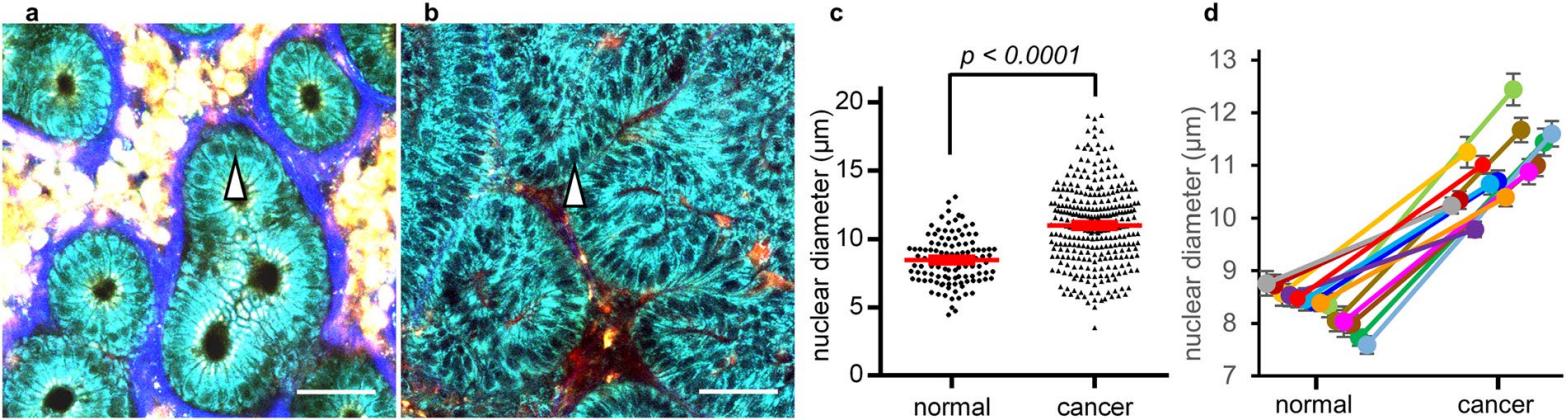
Evaluation of nuclear diameters in MPM images. (**a**,**b**) MPM images of normal colorectal mucosa (**a**) and colorectal cancer tissue (**b**). The samples in (**a**) and (**b**) were from the same patient. The nuclei in epithelial cells were identified as signal-void regions in the cytoplasm (arrow head). Bar: 50 µm. (**c**) Comparison of nuclear diameters between normal mucosa (Fig. 4a, *n* = 112) and cancer tissue (Fig. 4b, *n* = 297). The red line indicates the mean ± standard error of the mean (SEM). (**d**) Mean nuclear diameters in normal tissue (left) and lesion tissue (right). Dots of the same color with the line indicate the same patient (*n* = 14). Statistically significant differences were observed in all the patients analyzed. Error bars indicate ± SEM.

An advantage of fluorescent images is the ability to perform quantitative analyses. Unlike bright-field images of HE staining, fluorescent signals can be measured and quantified relatively easily^6–8^, leading to the identification of several parameters characterizing the images. Thus, we sought to extract useful numerical parameters from the NL-MPM images to distinguish cancer from normal features. First, enlarged nuclei were observed frequently in epithelial cells of cancer tissue, in comparison with normal tissues (Fig. 4a and b). Thus, we calculated and compared the nuclear diameters of epithelial cells in both normal tissue and cancer lesions of the same patients with the same size (a square region of interest [ROI] with a side length of 250 μm). As shown in Fig. 4c, nuclear diameters in cancer tissues were significantly larger than those in normal colorectal mucosa. Such significant differences were observed in all 14 patients analyzed (Fig. 4d).

### SHG signal from basement membrane is another numerical parameter to classify colorectal disease

**Figure 5:**
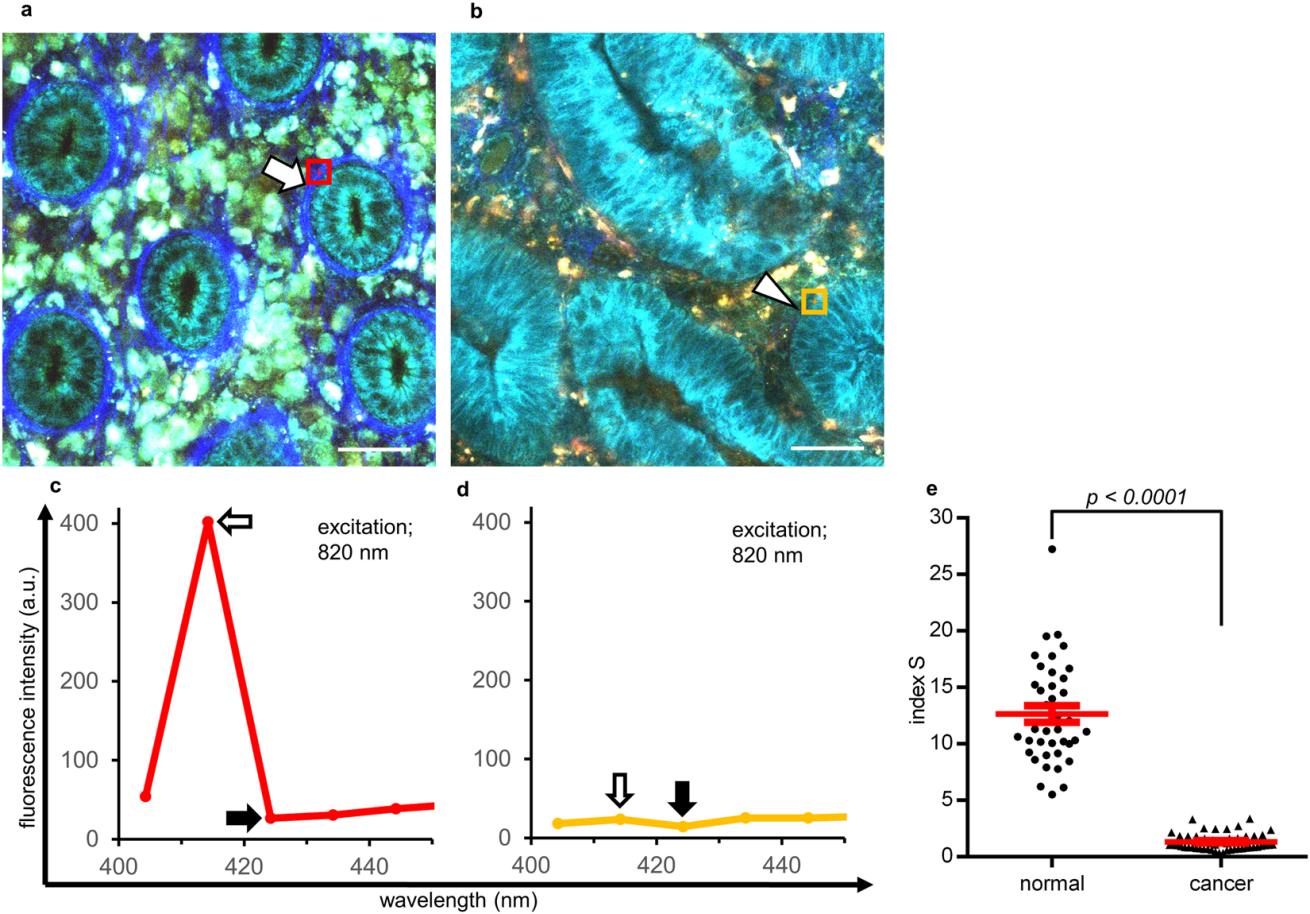
Evaluation of SHG signals from basement membranes with index S. (**a**,**b**) MPM images of normal colorectal mucosa (**a**) and cancer tissue (**b**). While the SHG signal was observed clearly in normal mucosa (arrow), regions in which the SHG signal was unclear or lost were observed in cancer lesions (arrow head). Bar: 50 µm. (**c**,**d**) Spectral analyses of regions with a clear SHG signal (**c**, red square ROI with a side length of 15 µm in (**a**)) and unclear signal (**d**, yellow ROI in (**b**)). Index S was calculated by dividing the fluorescence intensity at 410–420 nm (white arrow) by that at 420–430 nm (black arrow) in the square ROI under 820 nm excitation. (**e**) Index S of regions with a clear SHG signal in normal mucosa (left, *n* = 39) and unclear signal in cancer tissue (right, *n* = 51). Index S was calculated at three different regions per image in the spectral analysis. The red line indicates the mean ± SEM.

Second, one of the most distinguishing features of NL-MPM imaging is the visualization of collagen fibers in the basement membrane lining beneath the ductal structures, because the fibers provide SHG signals. Thus, we quantitatively evaluated the relationship between SHG fiber structures and colorectal disease. In normal colorectal mucosa, SHG signals from the basement membranes were observed in all cases analyzed (Fig. 5a), whereas such signals in corresponding areas were diminished in cancer tissues in 95% (19/20) of the patients analyzed (Fig. 5b). To quantify SHG signals, we calculated an index (‘index S’) using a spectral pattern analysis between 400 and 450 nm under excitation at 820 nm. The SHG signal was seen as a strong and narrow peak at 410–420 nm, but there was almost no emission signal at 420–430 nm or longer wavelengths. The fluorescence intensity ratio at 410–420 nm to that at 420–430 nm, index S, was high when the SHG signal was strong (Fig. 5c and d). We calculated index S from three independent regions per image using square ROIs with a side length of 15 μm. As shown in Fig. 5e, index S was high in normal tissue where fiber structures generating SHG signals were prominent but lower in the cancer lesion where SHG signals were lost. These findings indicate that loss of SHG signals from the basement membrane region could be an important characteristic of cancer tissue in the colorectal mucosa.

### Classification by two indices enables to distinguish NL-MPM images to normal and cancerous tissues quantitatively

**Figure 6:**
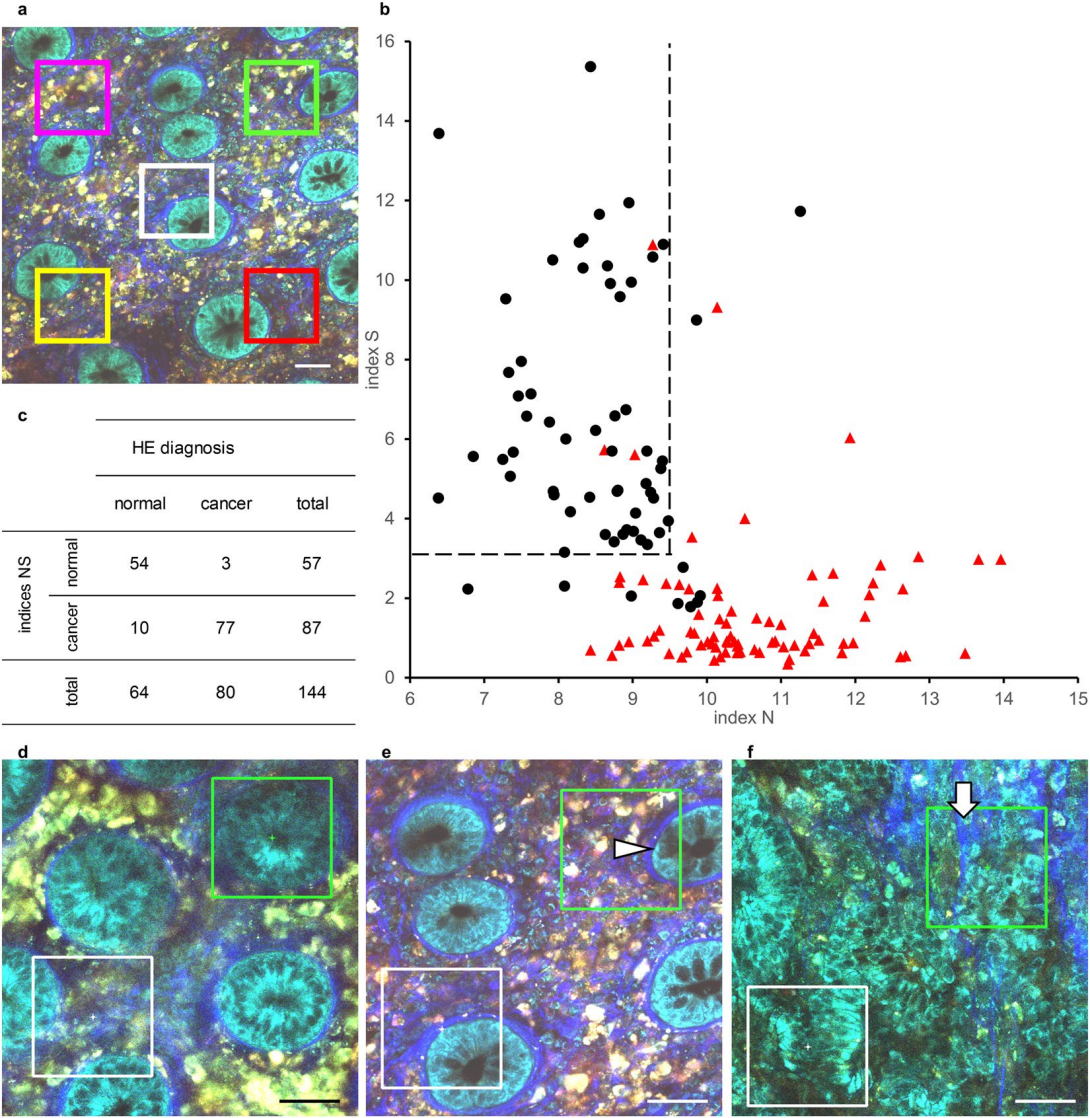
Classification analysis of MPM images from normal and cancer tissues using the two indices N and S. (**a**) Schematic overview of the areas selected for classification analysis with indices N and S. Five areas (square ROI with a side length of 100 µm) were determined in advance in the *x*-*y* coordinate plane for the original image files of the spectral analysis cases. Areas that contained no epithelial cell nuclei in the ROI (e.g., the pink ROI) were omitted from the analysis. Bar: 50 µm. (**b**) Scatter plot from the classification analysis using indices N and S. Final diagnoses of normal (black dot, *n* = 64) or cancer tissue (red triangle, *n* = 80) were made using HE-stained sections from the same specimens imaged by MPM. Areas that showed an index N ≤ 9.5 and index S > 3.1 (upper left area of the dashed line) were deemed normal in the classification analysis. (**c**) Classification result comparing HE diagnosis and criterion by indices N and S. (**d**–**f**) Representative images classified as false-positive (**d** and **e**) and false-negative results (**f**). Green ROIs represent the misclassified areas. The arrow head in (**e**) shows mucus in a goblet cell. The arrow in (**f**) shows fibrosis in the tumor. White ROIs represent correctly classified areas. Bar: 50 µm.

Next, we evaluated the utility of two quantitative parameters defined above, nuclear diameters (index N) and intensity of fibrous SHG signals (index S), as diagnostic tools for distinguishing normal and malignant lesions in colorectal mucosa. To make the evaluation more objective, avoiding manual artifacts and biases, five areas (square ROIs with sides of 100 µm each) were determined automatically in advance in the *x*-*y* coordinate plane for all original image files of the cases used for spectral analysis (Fig. 6a). Any area that contained no epithelial cell nuclei within the ROI was omitted from the analysis. Index N was defined as the mean nuclear diameter in the square ROI. Index S was calculated as described above. HE-stained sections from the same specimens used for MPM imaging were used to confirm the diagnosis. In total, 64 areas from normal tissue and 80 areas from cancer lesions were analyzed in terms of the N and S indices (Fig. 6b). Of them, 54 (84%) areas from normal tissues showed low index N and high index S values. In contrast, 61 (76%) areas from cancer lesions showed high index N and low index S values. From these distribution patterns, we defined normal tissue as that showing an index N ≤ 9.5 and an index S > 3.1 as thresholds. A lesion not meeting these criteria was deemed to be cancerous. According to these cut-offs, 54 of 64 areas were classified as normal, whereas 77 of 80 areas were classified as cancerous (Fig. 6c). The sensitivity and specificity of indices N and S were 96% and 84%, respectively. A malignant diagnosis based on indices N and S had a positive predictive value of 89%, and a normal diagnosis had a negative predictive value of 95%. The kappa coefficient between the HE-based diagnosis and the two indices was determined to be 0.82.

We then sought to identify the causes of the false-positive and false-negative results in the classification based on indices N and S. One of the most important factors that caused a false-positive result was blurred images, which resulted in low SHG signals (reducing index S) and difficulty in recognizing the shapes of small nuclei (increasing index N; Fig. 6d). Other possible factors causing false-positives included the presence of goblet cells (Fig. 6e). Specifically, mucus in goblet cells was recognized as a signal-void region against the background of a fluorescent cytoplasm, thus resembling nuclei. The most important factor associated with false-negative results was fibrosis, observed irregularly in tumors and leading to an inappropriate increase in index S (Fig. 6f).

As a result, we concluded that using the MPM imaging procedure, we could distinguish normal tissues and cancer lesions in fresh, human unstained colorectal mucosa quantitatively, in contrast to traditional pathological diagnosis, using the two indices N and S. However, there is still a need to improve the method to overcome the factors causing false-negative and false-positive results for more accurate classification.

## Discussion

NL-MPM has several advantages in visualizing unfixed thick tissues *in vivo*, using multiphoton excited auto-fluorescence and non-linear optical signals, such as SHG^9^. The goal of this study was to exploit these features to establish a novel diagnostic procedure for distinguishing normal tissue and cancer lesions, based on MPM techniques. For the clinical use of MPM techniques, it is desirable to avoid or minimize any need for fluorescent labeling, and thus the NL-MPM procedure appears quite suitable for clinical applications.

Other than NL-MPM, there are other methods of imaging living tissues that do not require labeling, such as Raman spectroscopy and photoacoustic microscopy. Raman spectroscopy relies on the vibrational properties and distribution of components, such as lipids, proteins, and DNA, to generate contrast^10^. Previous studies have indicated the value of label-free detection by Raman spectroscopy for guiding the surgical margin of brain tumor surgery^11, 12^, although the images are not very clear, and the depth of the imaged regions is currently limited^13^, compared with fluorescent microscopy. Photoacoustic microscopy is also considered a promising tool, based on a hybrid technique of optical excitation and ultrasound detection, and has been shown to be useful in the analysis of, for example, mouse brain^14^ and human samples^15^. However, given its high spatiotemporal resolution, advanced fluorescent imaging techniques, such as NL-MPM, seem advantageous for future application in clinical medicine.

There are many advantages to the NL-MPM technique described here over the ‘conventional’ diagnostic method based on HE staining. First, analysis by NL-MPM does not require excision or staining of tissues and allows rapid analysis of tissues. Thus, analysis by NL-MPM will be useful not only for histological diagnoses but also rapid intraoperative diagnoses, such as determination of surgical margins and identification of metastasis in regional lymph nodes. Another advantage of NL-MPM imaging is SHG-based visualization of the basement membrane lining beneath ductal structures. The basement membrane is hardly visible by HE staining alone, and immunohistochemical staining is typically necessary to evaluate basement membrane characteristics. Moreover, several reports have indicated that breakdown of the basement membrane occurs in colorectal carcinoma tissue^16, 17^. The SHG signal is produced only by tightly aligned collagen molecular structures, and it scales quadratically with the density of such aligned molecules^18^. Our results indicated that tightly aligned, well-organized collagen structures exist in the basement membrane of normal colorectal mucosa, whereas these organized structures are lost in cancer tissues. Using NL-MPM, we obtained important information about the basement membrane status in cancer lesions without the need for staining. Third, the NL-MPM technique allows identification of normal and malignant lesions quantitatively in terms of numeric parameters, which has hardly been attempted using conventional HE staining. Quantitative analyses using numeric indices also allow automated classifications using computer systems. Computer-aided diagnosis techniques have advantages in interpreting and classifying digital data quantitatively with a reduced need for human resources. Today, many clinical pathologists spend much of their time diagnosing and classifying malignant neoplasm specimens as a result of increased incidence. The demand for subtyping malignant tumors based on their biological and genetic properties will further increase pathologist workloads, requiring immunohistochemical staining and genetic analyses. The adoption of new diagnostic tools, including the NL-MPM technique, will provide advantages to pathologists in terms of reducing workloads, providing useful information for diagnosis.

We identified the autofluorescent molecules responsible for NL-MPM imaging of human colorectal mucosa as NAD(P)H and FAD based on spectral analyses. However, the possibility that other substances are also involved in this autofluorescence cannot be excluded. Some plausible candidates are lipofuscin and lipofuscin-like compounds, which are residual aggregated substances of undigested cell material^19^. In fact, some immune cells such as macrophages and eosinophils have been reported to contain lipofuscin and lipofuscin-like lipopigments^20^. Moreover, lipofuscin has a broad-spectrum emission profile overlapping that of FAD, because the heterogeneous composition of lipofuscin exhibits various emission properties^21^. Although no obvious findings were evident in our HE-stained samples, melanosis-related products can also be responsible for autofluorescence^22^. Future studies using accurate spectral analyses under diverse excitation/emission conditions are necessary to confirm that the emissions result from FAD.

Regarding gastrointestinal diseases, including colorectal carcinoma, the development of endoscopic techniques using MPM systems is also important for the clinical application of NL-MPM. If we can develop and use endoscopy with MPM systems, real-time diagnosis at high resolution would be possible during routine endoscopy. Moreover, this device has benefits in the field of endoscopic resection, such as endoscopic mucosal resection and endoscopic submucosal dissection, because prompt determination of surgical margins may be facilitated by NL-MPM imaging. There have already been several attempts to develop a compact endoscope using a MPM system^23^; thus, the clinical use of NL-MPM is becoming a reality. An obstacle with applying endoscopy with MPM is the need to fix the field of view against the movement of tissues during observation. This did not need to be considered in this study, because we used removed colorectal tissue. Appropriate suction in the endoscope and the development of a useful adapter to ensure the visual field may solve this issue.

Currently using our MPM imaging device, we can only evaluate the lamina propria mucosae of the colorectum (at a typical depth of <120 µm). We could not obtain clear images at greater depths. Image data from deeper regions, such as around the muscularis mucosae and submucosa, contain valuable information, such as progression status of the disease, invasion depth, and presence/absence of vascular invasion^24, 25^. Moreover, some malignant tumors are difficult to detect by analysis of the mucosal surface, such as scirrhous gastric carcinoma, which often infiltrates beneath the mucosal layer^26^. Thus, more work to acquire images from deeper areas is needed to further develop MPM imaging technique as a diagnostic method. Challenges in developing such devices include improvement of objective lenses suitable for observation at greater depths and introduction of new detector systems, such as those with adaptive optics.

Although we analyzed colorectal diseases, NL-MPM should be effective for diagnoses in other tissues and organs. It has already been reported to be effective for diagnoses and therapeutic assessments in skin^27–29^. Other organs in which an endoscopic approach is appropriate, such as the respiratory tract, urinary system, and gynecological organs, will become important targets with the development of new devices. Moreover, even if it is difficult to approach from the surface, target organs for rapid intraoperative diagnoses, such as lymph nodes and organs in which minimal invasion is desirable, including the brain, will be also suitable for NL-MPM-based analysis. When considering the utility of NL-MPM analyses, thorough evaluation of the distribution of auto-fluorescent substances is also important, as well as collagen molecules that produce SHG signals in the organ of interest.

## Methods

### Clinical specimens

Samples of colorectal mucosa were collected from 22 patients with colorectal carcinoma after surgery at Osaka University Hospital. Of these patients, both normal and tumor colorectal mucosal tissues were collected from 14, while only tumor tissues were collected from 6 and only normal mucosa tissues from 2 patients. All patients were diagnosed histologically before surgery by endoscopic biopsies. Samples for imaging were collected from the edge of tumors or from residual normal mucosal tissue around the tumors. The collected samples were immediately delivered to the room used for MPM imaging using phosphate-buffered saline and wet paper. Immediately after imaging, samples were fixed in 10% neutral buffered formalin (Muto Pure Chemicals, Tokyo, Japan) and processed routinely for paraffin embedding. All patients provided written informed consent, in accordance with the ethics committee requirements of Osaka University and with the Declaration of Helsinki. This study was conducted under the supervision of the ethics board of Osaka University Graduate School of Medicine. The Osaka University Graduate School of Medicine Institutional Review Board approved this study protocol on December 17, 2015 (No. 15369).

### MPM imaging of human colorectal tissues

MPM imaging was performed using protocols described previously^30^ with modifications. Briefly, the imaging system consisted of an upright multiphoton microscope (A1RMP+, Nikon) driven by a laser (Chameleon Vision II Ti: Sapphire; Coherent, Inc.) tuned to 780 nm, and an upright microscope equipped with a ×25 water-immersion objective (CFI75 Apo 25 × W MP/NA 1.10, Nikon). The microscope was enclosed in an environmental chamber. Colorectal tissue samples were placed on a rubber plate with the mucosal surface facing upwards with small pins and overlaid with a coverslip to form a drop of water between the sample and objective lens. To detect multiphoton excited fluorescence and SHG emission signals, 417/60 nm, 480/40 nm, and 629/56 nm band-pass filters were used. Raw imaging data were processed using the NIS-Elements software (Nikon). Minor image processing steps, such as brightness adjustments, were also performed using Photoshop for better visualization. Nuclear diameters were evaluated by measuring the major axes of signal-void regions in epithelial cells present in a square ROI using the NIS-Elements software. Index N was defined as the mean nuclear diameter in the square ROI. Spectral analyses were performed in 18 patients (normal and cancer lesions in 12, cancer lesions only in 5, and normal tissue only in 1). To collect data, we used the spectral detector of the A1RMP + microscope (Nikon). Tissues were imaged to record emission spectra from 400 to 650 nm (collected in 25 bins, each ~10 nm wide) under a predetermined wavelength excitation. Each fluorescence intensity was recorded at 4,096 gray intensity levels (12 bit). Index S was defined as the ratio of the fluorescence intensity at 410–420 nm to that at 420–430 nm in the square ROI under excitation at 820 nm.

### Histology and immunohistochemistry

Paraffin-embedded specimens were cut to 4 μm sections and stained using immunoperoxidase-based procedures. After antigen retrieval using a Pascal pressurized heating chamber (DAKO A/S, Glostrup, Denmark), the sections were incubated with DAKO REAL Peroxidase-Blocking Solution to inactivate endogenous peroxidases, incubated with the indicated antibodies, and then treated with the ChemMate EnVision kit (DAKO). DAB (DAKO) was used as the chromogen. Sections were counterstained with hematoxylin for 1 min before mounting. The primary antibodies used included anti-FAD (PAG408Ge01, Cloud-Clone Corp.) and anti-laminin (ab11575, Abcam). A rabbit polyclonal antibody (Biolegend) was used as an isotype control. Sections were also stained with HE using a standard protocol.

### Statistical analysis

Welch’s t-test was used for comparisons between groups with Gaussian-like distributions. For highly skewed distributions, the Mann-Whitney U-test was used to calculate p-values. To compare the diagnostic accuracies between conventional diagnosis by HE staining and classification by indices N and S, the kappa coefficient was calculated as a measure of interobserver agreement.
